# Residents’ identification of learning moments and subsequent reflection: impact of peers, supervisors, and patients

**DOI:** 10.1186/s12909-020-02397-7

**Published:** 2020-12-02

**Authors:** Serge B. R. Mordang, Eline Vanassche, Frank W. J. M. Smeenk, Laurents P. S. Stassen, Karen D. Könings

**Affiliations:** 1 Department of Educational Development and Research, School of Health Professions Education, Maastricht University, P.O. Box 616, 6200 MD Maastricht, The Netherlands; 2 Centre for Innovation and the Development of Teacher and School, University of Leuven, Leuven, Belgium; 3 Department of Educational Development and Research, School of Health Professions Education, Maastricht University, and Catharina Hospital Eindhoven, Maastricht, The Netherlands; 4 Department of Surgery, Maastricht University Medical Center, Maastricht, The Netherlands

**Keywords:** Reflection, Workplace learning, Postgraduate medical education, Safe learning environment, Social context

## Abstract

**Background:**

The clinical workplace offers residents many opportunities for learning. Reflection on workplace experiences drives learning and development because experiences potentially make residents reconsider existing knowledge, action repertoires and beliefs. As reflective learning in the workplace cannot be taken for granted, we aimed to gain a better insight into the process of why residents identify experiences as learning moments, and how residents reflect on these moments.

**Methods:**

This study draws on semi-structured interviews with 33 medical residents. Interviews explored how residents identified learning moments and how they reflected on such moments, both in-action and on-action. Aiming for extensive explanations on the process of reflection, open-ended questions were used that built on and deepened residents’ answers. After interviews were transcribed verbatim, a within-case and cross-case analysis was conducted to build a general pattern of explanation.

**Results:**

The data analysis yielded understanding of the crucial role of the social context. Interactions with peers, supervisors, and patients drive reflection, because residents want to measure up to their peers, meet supervisors’ standards, and offer the best patient care. Conversely, quality and depth of reflection sometimes suffer, because residents prioritize patient care over learning. This urges them to seek immediate solutions or ask their peers or supervisor for advice, rather than reflectively deal with a learning moment themselves. Peer discussions potentially enhance deep reflection, while own supervisor involvement sometimes feels unsafe.

**Discussion:**

Our results adds to our understanding of the social-constructivist nature of reflection. We suggest that feelings of self-preservation during interactions with peers and supervisors in a highly demanding work environment shape reflection. Support from peers or supervisors helps residents to instantly deal with learning moments more easily, but it also makes them more dependent on others for learning. Since residents’ devotion to patient care obscures the reflection process, residents need more dedicated time to reflect. Moreover, to elaborate deeply on learning moments, a supportive and safe learning climate with peers and supervisors is recommended.

## Background

Reflection on experiences in everyday work is of vital importance for learning [1–3], particularly for residents, who in the beginning of their medical career have many new and unfamiliar workplace experiences. Reflection on such experiences allows residents to improve their knowledge, skills, and attitudes, and provides a sound foundation for continuing professional development and life-long learning [4–6]. By frequently reflecting, residents broaden and deepen the action repertoire they use in the workplace [5, 7, 8]. Argyris [9] defines this process as a “reflexive loop”, highlighting the circular process of constantly rethinking and adjusting one’s actions, and the knowledge and beliefs on which these actions are based. Yet, reflection does not occur automatically [10]. To better understand reflection on workplace experiences, it is important to learn more about the initiation of residents’ reflection process and the process itself.

Reflection is defined as the process of letting “future behaviour be guided by a systematic and critical analysis of past actions and their consequences” [11](p. 827). Reflection begins when residents identify a workplace experience as a learning moment that can influence their professional behavior [7, 12]. Learning moments are essential to make residents aware of existing flaws, forcing them to revisit their knowledge, beliefs, and action repertoire [13]. It is not without reason that such learning moments form part of all major reflection theories, albeit in many guises, ranging from critical incidents [14], puzzling events [8], significant or memorable and vivid happenings [15], to concrete experiences [16], and influencing events [17]. Furthermore, extensive research on teacher professional development demonstrated the importance of learning moments in reflective learning (see e.g. [18]). However, it is unclear what triggers residents to identify some workplace experiences as learning moments, while other experiences not as such. This understanding would be crucial for meaningfully supporting residents in the initiation of reflection in the workplace. Therefore, our first research question concerns why residents identify workplace experiences as learning moments.

A model that is frequently applied to the theory and practice of workplace learning in health professions education is the reflection model by Schön [3, 7, 19]. Schön’s model breaks reflection down into two major phases, specifically “reflection-in-action” and “reflection-on-action.” Reflection-in-action occurs instantly, by thinking on the spot, in response to a non-routine or unfamiliar experience that is typically accompanied by a moment of uncertainty, perplexity, or confusion. As a result, an inner drive arises to examine and challenge current behavior in practice and the knowledge and beliefs underlying the behavior. Reflection-on-action [7] takes place later when thinking back to the workplace experience, in order to explore behavior in response to the experience and to discover why prior knowledge and beliefs were insufficient to deal with the experience. This process leads to new understandings and possibilities for actions in similar future situations. Through reflection in-action and on-action, residents learn and develop new skills, deepen existing skills, and learn to understand why they act the way they do in workplace situations. The process of reflection-in-action and on-action can differ across contexts and individuals [3]. Linked to the identification of learning moments, our second research question concerns how residents reflect-in-action and on-action on workplace experiences and what may facilitate or hinder these processes.

## Methods

### Context

This study follows up on a previous study in which Könings et al. [20] quantitatively compared the reflection process between residents who received either a smartphone app to facilitate easy capturing of learning moments in the workplace or coaching sessions aimed to deepen reflection on learning moments, or they received both, or none. It appeared that smartphone app users captured more and reflected on more learning moments and that participants in coaching sessions were more alert to and pursued more learning moments. To answer our current research questions, we did a follow-up study a few weeks afterwards (in December 2012) where we interviewed part of these medical residents. Interview data were subsequently submitted to a within-case (on an individual, participant basis) and cross-case (across all participants) analysis to build a general pattern of explanation, overarching the different types of reflection support.

### Participants

From the total sample in the previous study (*N* = 64), KK purposefully sampled eight or nine residents from each reflection support groups. In total, half of the residents (*N* = 33, 18 males and 15 females) participated in the semi-structured interviews. Of the residents, seven were in their first year, 11 in their second year, three in their third year, seven in their fourth year, and five in their fifth year of training. Residents were from different medical specialties: eight from internal medicine, five from orthopedics, two from anesthesiology, dermatology, pediatrics, pulmonology, surgery, and radiology, and one from cardiology, gynecology, clinical genetics, ear nose and throat, neurology, ophthalmology, rheumatology, and urology. Residents were employed at two hospitals in the south of the Netherlands.

### Materials

Semi-structured interviews explored how residents identified learning moments. Questions invited residents to give a detailed account of a moment that had significantly impacted their learning and a moment that lacked such impact and why they thought this was so. In addition, interviews explored how residents processed learning moments, concerning how they reflected both in-action and on-action. To obtain “thick descriptions” [21] of reflection, consisting of subjective explanations and meaning, all questions were open-ended, building on and deepening residents’ answers. Example questions were ‘Can you think of any learning moment you encountered?’, ‘What makes such a moment a learning moment for you?’ and ‘What did you subsequently do with this learning moment’. See Appendix A for an overview of the interview questions and their link with the research questions.

### Procedure

To encourage residents to participate in the complete research project (including the current interview study), all participants were awarded a training certificate upon full completion of both studies. In the hospitals where these residents are trained, residents are required to earn several of such certificates per year. While not playing a role in participants’ training program, KK conducted the interviews, which lasted approximately 30 min. To ensure anonymity, codes were used for each participant in the analysis and reporting of data, which de-identified all participant data. The Netherlands Association for Medical Education approved the study (no. NERB0031) before launch. All residents signed an informed consent prior to participation.

### Data analysis

All interviews were recorded and transcribed verbatim. Transcriptions were entered into MAXQDA, a qualitative data analysis program. Data analysis consisted out of deductive coding, for which we used the framework of Schön (learning moments, reflection-in-action, reflection-on-action) [7]. SM, EV and KK first familiarized themselves with the data by reading it multiple times, before dividing the transcripts into smaller text segments that each constituted one coherent message or meaningful stance from participants (varying in length from a couple of words to a short paragraph). In an iterative process, interpretative codes (reflecting the theoretical framework) were used to code each text segment. For a systematic within-case analysis [22] SM wrote a summary of the data of each participant in relation to the research questions, including: how the participant identified learning moments, as well as how the participant reflected in-action, and on-action. The summaries served the purpose to structuring the large amount of data and creating a better workable cross-case analysis, while preserving the richness of the data by including direct quotes from the interviews. In a second step, the cross-case analysis build an overall understanding and general pattern of explanation how residents identify learning moments, and reflect-in-action and on-action [22]. Analyzing involved constantly moving back and forth between all summaries, that described answers to the research questions at the level of the individual participant, and synthesizing these to the overall answers to our research questions (constant comparative method [23]). SM, KK, and EV discussed findings until consensus occurred, thereby warranting the results.

## Results

Results revealed multiple ways by which the social context impacted how residents came to identify learning moments and reflected in-action and on-action.

### Identification of learning moments

Residents more likely identified workplace experiences as learning moments in the presence of peers and supervisors, for example during handoffs, formal schooling, and informal conversations in the workplace, rather than when the experience took place while working on their own. With regard to peers, identification of learning moments happened when residents realized that peers held different ideas about possible treatments or possessed different or deeper knowledge. Residents seemingly felt pressure to measure up to their peers and the standards implicitly set by them. In the words of a resident (R1):

“I certainly do have those [learning moments] during handoffs too … , because then I notice, like, that others can produce it really easily and then indeed, oops, I don’t know that … Shouldn’t I have known that too?”

Feelings of being less knowledgeable than their peers, triggered the need to remediate perceived discrepancies, and hence stimulated residents to identify these experiences as learning moments.

In a similar way, interactions with supervisors stimulated experiences to be seen as learning moments. Residents often identified a mistake as a learning moment. Explicit notion by supervisors reinforced residents’ perceived urgency of the learning moment, effectively fueling reflection:

It came out last week, that I gave the wrong dose of medicine … That does leave you uncomfortable for a while … A mistake is always a learning moment that impacts you, of course … But it is still unpleasant and especially so because my supervisor noticed that. (R3).

In addition to peers and supervisors, patient care was crucial for the identification of learning moments. Since providing high-quality patient care was residents’ number one priority, patient contacts were often a major source to identify such moments:

“You want to do it right for that patient, make the correct diagnosis, the right treatment, that is.” (R11).

Regarding the nature of the learning moments, residents regularly identified experiences involving medical competencies as learning moments. In such learning moments, residents were often immediately aware of the problem at hand (e.g., “I don’t understand this syndrome at all” (R4)). Experiences linked to the awareness of a deficiency in general competencies, such as communication, patient safety, or ethics, however, were often not identified as learning moments at the time residents encountered them.

“General competencies, those are difficult, because you don’t always consciously think about them the moment they happen, but when you start to think back, you think, wait a minute, that is not right.” (R7).

The identification of learning moments from experiences involving general competencies occurred often at a later time, for example when writing physician notes, or contemplating a workday. Furthermore, residents mention that identification of learning moments changes over time: they identify more learning moments involving both medical and general competencies in the beginning of their residency, while they identify less learning moments involving general competencies when being more experienced in their residency.

“as long as you are a freshman you just have hundreds of learning moments*.*” (R23).

### Reflection-in-action and reflection-on-action

Following the identification of the learning moment, residents reflected in-action and on-action. Reflection-in-action can be hindered because of time constraints, causing that residents need to find adequate solutions quickly. In such cases, when confronted with a learning moment, residents often enlisted the help of peers and supervisors:

“We have one supervisor sitting at the end of the hallway and that’s where you walk over to the moment you don’t know.” (R3).

While instant advice can facilitate effective dealing with a clinical situation, residents were less inclined to reflect on these learning moments themselves. As one resident pointed out:

“that I already asked someone before: How about that? And when I receive a short answer, I no longer go and look it up myself.” (R27).

In the process of reflection-on-action, residents frequently pointed to the facilitating role of their peers. They perceived the opportunity to consult their peers, thereby discussing different ways to pursue learning moments, as very valuable, especially when a safe environment for discussion was available and when learning moments involved general competencies.

“In itself I think it is useful to sometimes hear, and especially when it comes to non-medical subject matter, how other residents deal with it or how they handle it.” (R22).

Such perceptions differed when it involved supervisors, however. In their presence, residents reported to sometimes feel restrained or judged, preventing them from freely discussing and sharing thoughts and ideas on learning moments. Supervisor involvement can potentially hinder discussing learning moments openly, due to a perceived less safe learning environment:

If my professor had sat there, who is also my instructor, yes, then you sometimes would have had to guard your speech. He won’t hold it against you, because he is a very good instructor, but still you enter such a coaching session differently. (R14).

Residents were strongly committed to patient care, which seems to counteract effective reflection-in-action and reflection-on-action. Not determining the right treatment could have direct consequences for patients, while residents’ failure to reflect on one’s own competencies would not immediately. Therefore, when both priorities were in conflict, residents tended to favor patient care. Such conflicts often occurred, as residents had to see a significant number of patients each day, which increased the need for quick solutions. This came at the expense of deeper reflection as the following statement reveals:

“Shoot, where does it go wrong with that patient?... I’ve had little time recently to really look up many things, so I have not done any real look-up stuff.” (R14).

Furthermore, as residents considered a learning moment sufficiently dealt with when a patient was treated, reflection on learning moments at a later time to improve action patterns for similar future situations was often absent. Hence, important lessons to be learned from these moments were possibly neglected.

“so I looked something up and I know I looked it up and then three months or so later you run into it again and then you think, yes, shoot, what was it again?” (R29).

With respect to learning moments concerning general competencies, residents’ reflection seldom took place “in-action”. When residents encountered such experiences, they did not regard them as sufficiently urgent to require deep, on-the-spot reflection. Rather, residents thought about these learning moments later, when reflecting-on-action at the end of the working day. As a result, reflection-in-action was impeded and often incomplete:

Even if communication is not optimal perhaps or you have another issue, that does not usually prevent you from doing your job … in the evening or so I thought, ‘oh yes, that is actually also something to think about’ or … somebody [a peer] said something else, I suddenly realized, like, ‘oh yes, I recently experienced a similar situation and indeed I did not quite know, uh, what to do with it either’. (R6).

## Discussion

Concerning research question 1, our study shows that residents identified workplace experiences as learning moments because of the perceived importance of such experiences during interactions with peers, supervisors, and patients in their workplace environment. They did so, by identifying these moments through interactions with peers and supervisors at (in) formal moments in the workplace and through patient care. While all these stakeholders may aid identification of learning moments, peers, supervisors, and patients also support or hinder residents´ subsequent reflection on learning moments. Concerning research question 2, residents reflected on the spot, often using the expertise of peers and supervisors to immediately deal with the learning moment at hand. Afterwards, residents stress the importance of discussing their learning moments with their peers.

Regarding the identification of moments for reflection and learning, residents identified learning moments when they interacted with peers and supervisors, stimulated by their ambition to meet their supervisors’ standards and measure up to their peers. Residents wanted to show their best and leave a good impression in front of their peers and supervisor. This might on the one hand enhance their reflective learning but may on the other also induce peer pressure and stress. The striving for optimal performance could be due to the demanding work environment [24, 25] that produces feelings of self-preservation and image management [26], and a desire to becoming a member of the community in the workplace [27]. Daily patient contacts, too, were an important source and facilitator of identifying learning moments, because residents wanted to provide the best care possible to their patients. Altruism and the desire to help others are well-known prime intrinsic motivators throughout postgraduate medical training [28]. In setting their learning agenda, residents tend to focus externally.

As for reflection-in-action, support from peers and supervisors helped residents to deal with learning moments more easily on the spot. These ‘quick fixes’ made residents rely on others for responding to learning moments in the clinical practice, indicating a dependency on their social context. Hence, it may also discourage residents to seek and think of solutions themselves, limiting residents to shape their own learning. The high prioritized patient care, with its existing time constraints [29], possibly caused by a growing patient population [30], long waiting lists [31] and a sharp focus on productivity, compounds this situation further by leaving little time for reflection. Additionally, residents’ intrinsic motivation to help and attend to patients sometimes limited their opportunity for reflective learning from patient contacts. As the practice of treating patients may hinder the quality and depth of reflection-in-action, possibly leading to superficial completion of the learning process, residents need to be granted dedicated time for reflection on learning moments identified during patient care.

For reflection-on-action, peers and, to a lesser extent, supervisors wielded the power to encourage residents to reflect on workplace experiences later, when a safe environment was offered. Receiving support from peers when reflecting deeply on learning moments reinforced residents’ feelings of acknowledgment and trust [32], facilitating to elaborate on experiences with each other. The presence of their supervisor, however, sometimes invoked anxiety and thus hindered deep reflection, as residents’ sense of self-preservation and image management discouraged them to elaborate. Hence, in order for residents to freely express themselves and learn from potential failures, psychological safety must be guaranteed [33, 34]. An independent coach or supervisor could support such an environment [20, 35–37]. Furthermore, supervisors may benefit from a training to enhance their competency for reflective supervision [38].

That said, our findings extend to existing models of reflection, social learning, and workplace learning in medical education [7, 12, 39, 40] by demonstrating how the social context affects residents’ initiation of their reflection processes in different ways. The current study shows that the complex social context of residency training, offering interactions with peers, supervisors, and patients, not only helps residents to identify distinct medical and general workplace experiences as learning moments, but also shapes their reflection process. While social learning theories, for instance social cognitive theory [41] transformative learning [42] and recent work on experiential workplace learning [43], suggest that learning occur through the observation and imitation of others, our study shows that peers and supervisors can indeed help residents to resolve these moments effectively and efficiently, therefore enhancing reflection by offering a supportive learning environment. Conversely, however, all aforementioned key actors can also hinder the reflection process, for instance, by decreasing self-initiative, not ensuring a psychological safe environment, or prompting a high demanding care agenda. Hence, reflection can indeed be considered as an interactive process situated in a social context [43–45], which presents both supporting and inhibiting forces.

### Practical implications

Our study has several practical implications. First, residents need to be granted dedicated time for reflection on learning moments, as residents’ devotion to patient care tends to obscure learning needs. Second, in view of the apparent learning gains, residents should be encouraged to discuss and share their ideas on learning moments with peers, for example during handovers or reflective sessions, especially when such moments concern general competencies. Third, supervisors should balance between critically challenging residents to develop themselves and offering a psychological safe learning environment, for instance by allowing a certain degree of error for residents to make. They also have to find a balance between pointing out areas for improvement regarding knowledge and skills by giving ‘quick fixes’ and encouraging residents to self-direct their reflective learning.

### Limitations

In terms of limitations, we have to realize that the residents in our sample also took part in a previous study wherein most of them received different kinds of reflection support tools. Our sample may therefore differ from residents in regular settings. We considered it as a strength of our sample that these residents were potentially more conscious of reflection, which might have emboldened them to describe their thoughts more explicitly. It was beyond the focus of this study to explore possible differences in reflection processes of residents who earlier received different kinds of reflection support. Another limitation is that our explanation, describing the importance of self-preservation, image management, and psychological safety in the reflective learning process, predominantly highlights the perception of residents. Perceptions and intentions of supervisors, medical educators, or patients on residents’ reflective processes were beyond the scope of this study. To improve residents’ reflection process considering their perspectives, future research could investigate the perceptions of others, e.g. to understand why supervisors support and guide residents the way they do. Additionally, other theoretical or methodological approaches could be used to deepen our understanding of the mediating or moderating role that the social context has on residents’ reflecting and learning at the workplace. Observations, next to interviews, could form the base of such research. Analyzing these with a framing analysis, which reveals how interactions are interpreted by different actors, would help to understand how all key actors in residents’ social context experience interactions between each other [46] and how this impacts reflection. Furthermore, we only interviewed residents at one time-point during their training, neglecting personal learning over time. Hence, we invite future studies to investigate how residents learn new complex skills through reflection on a longitudinal scale, for example by exploring how interactions in residents’ social context develop over time.

## Conclusion

Identifying workplace experiences as learning moments is crucial for the initiation of the reflection process of residents. The social context in the workplace, consisting of peers, supervisors and patients promotes residents’ identification of learning moments and can thereafter facilitate but also hinder reflection on such moments in the workplace. Colleagues should keep residents sharp and focused in the workplace for noticing learning moments. To elaborate more deeply on learning moments, a supportive and safe learning climate with peers and supervisors is recommended.

## Data Availability

The Dutch datatranscipts collected during the current study are available from the corresponding author on reasonable request.

## Appendix

**Table 1:** Interview guide (with related research question between brackets)

In what situations did you recognize learning moments? What were your most important reasons to identify a learning moment? (RQ1) What made such a moment valuable or instructive for you? (RQ1) What did you do with the learning moments afterwards? (RQ2) What did you learn from them? If you did reflect on them furthermore, why not? (RQ2) How did the learning influence the future identification of the learning moments? (RQ1) Did you talk to anyone about them? With whom? (RQ2) *The resident was then asked to describe two learning moments that had a lot of impact and two moments that did not. About these learning moments the following questions followed, separately for both learning moments with and without impact:* What made you want to remember this situation? (RQ1) What made this situation into a learning moment for you? What were the conditions, what was your role? (RQ1) What were your thoughts at those moments? (RQ2) What were your later reflections on these learning moments? (RQ2) Did you learn from your reflection on the moments? If you did not reflect, why not? (RQ2) Did you talk with anyone about it? With whom? (RQ2) Did you change your behavior afterwards? (RQ2)	

Note: This guide provides an overall impression of the interview questions, as interviews were semi-structured. Residents’ answers affected follow-up questions that were asked

## Abbreviations

N: Number of individuals
R: Resident

## References

[1] Baernstein A, Fryer-Edwards K (2003). Promoting reflection on professionalism a comparison trial of educational intervention for medical students. Acad Med.

[2] Epstein RM, Hundert EM (2002). Defining and assessing professional competence. JAMA..

[3] Mann K, Gordon J, MacLeod A (2009). Reflection and reflective practice in health professions education: a systematic review. Adv Health Sci Educ Theory Pract.

[4] Branch WT, Paranjape A (2002). Feedback and reflection: teaching methods for clinical settings. Acad Med.

[5] Lyons N. Handbook of reflection and reflective inquiry: Mapping a way of knowing for professional reflective inquiry. Springer Science & Business Media; 2010.

[6] Aronson L (2011). Twelve tips for teaching reflection at all levels of medical education. Med Teach.

[7] Schön D (1983). The reflective practitioner.

[8] Dewey J (1933). How we think.

[9] Argyris C (1990). Overcoming organizational defenses.

[10] Driessen E, van Tartwijk J, van der Vleuten C, Wass V (2007). Portfolios in medical education: why do they meet with mixed success? A systematic review. Med Educ.

[11] Driessen E, van Tartwijk J, Dornan T (2008). The self-critical doctor: helping students become more reflective. Br Med Educ.

[12] Boud D, Keogh R, Walker D (1985). Turning experience into learning.

[13] Vachon B, Leblanc J (2011). Effectiveness of past and current critical incident analysis on reflective learning and practice change. Med Educ.

[14] Schön D (1987). Education the reflective practitioner.

[15] Brookfield S (1998). Critically reflective practice. J Contin Educ Heal Prof.

[16] Kolb DA (1974). Toward an applied theory of experiential learning.

[17] Boud D, Keogh R, Walker D (2013). Reflection: turning experience into learning.

[18] Tripp D (1992). Critical theory and educational research. Issu Educ Res.

[19] Mamede S, Schmidt HG (2005). Correlates of reflective practice in medicine. Adv Health Sci Educ.

[20] Könings KD, van Berlo J, Koopmans R, Hoogland H, Spanjers IA, ten Haaf JA, van der Vleuten CP, van Merrienboer JJ (2016). Using a smartphone app and coaching group sessions to promote Residents' reflection in the workplace. Acad Med.

[21] Geertz C. Thick description: Toward an interpretive theory of culture. Turning points in qualitative research: Tying knots in a handkerchief. 1973;3:143–68.

[22] Miles MB, Huberman AM. Qualitative data analysis An expanded sourcebook. Los Angeles: Sage; 1994.

[23] Glaser B, Strauss A (1967). Grounded theory: the discovery of grounded theory. J Br Socio Assoc.

[24] Dyrbye LN, West CP, Satele D, Boone S, Tan L, Sloan J, Shanafelt TD (2014). Burnout among U.S. medical students, residents, and early career physicians relative to the general U.S. population. Acad Med.

[25] Cohen MJ, Kay A, Youakim JM, Balaicuis JM (2009). Identity transformation in medical students. Am J Psychoanal.

[26] Hodges BD, Lingard L. In: Anderson MB, editor. The Question of Competence: Reconsidering Medical Education in the Twenty-First Century. Cornell University Press; 2013. https://digitalcommons.ilr.cornell.edu/books/84/.

[27] Sfard A (1998). On two metaphors of learning and the dangers of choosing just one. Educ Res.

[28] Ratanawongsa N, Howell EE, Wright SM (2006). What motivates physicians throughout their careers in medicine?. Compr Ther.

[29] Winkel AF, Hermann N, Graham MJ, Ratan RB (2010). No time to think: making room for reflection in obstetrics and gynecology residency. J Grad Med Educ.

[30] Kirch DG, Petelle K (2017). Addressing the physician shortage: the peril of ignoring demography. JAMA..

[31] Siciliani L, Moran V, Borowitz M (2014). Measuring and comparing health care waiting times in oecd countries. Health Policy.

[32] Tjosvold D, Yu Z-Y, Hui C (2004). Team learning from mistakes: the contribution of cooperative goals and problem-solving. J Manag Stud.

[33] Edmondson AC, Lei Z (2014). Psychological safety: the history, renaissance, and future of an interpersonal construct. Ann Rev Organ Psychol Organ Behav.

[34] Carmeli A, Gittell JH (2009). High-quality relationships, psychological safety, and learning from failures in work organizations. J Organ Behav.

[35] Sandars J (2009). The use of reflection in medical education: AMEE guide no. 44. Med Teach.

[36] Könings KD, Gijselaers WH. Bringing learning to the workplace: a smartphone app for reflection and increased authenticity of learning. In: Dailey-Hebert A, Dennis KS, editors. Transformative perspectives and processes in higher education. an eBook, Springer; 2015. p. 117–35.

[37] Daniel S, Lee AL, Switzer-McIntyre S, Evans C (2016). An innovative program to support internationally educated health professionals and their instructors: role of the clinical practice facilitator. J Contin Educ Heal Prof.

[38] Weiss EM, Weiss S (2001). Doing reflective supervision with student teachers in a professional development school culture. Reflective Pract.

[39] Moon JA. A handbook of reflective and experiential learning. London: Psychology press; 2004.

[40] Mezirow J (1991). Transformative learning: theory to practice.

[41] Bandura A (2001). Social cognitive theory: an agentic perspective. Annu Rev Psychol.

[42] Mezirow J (1997). Transformative learning: theory to practice. New Direction For Adult and Continuing Education.

[43] Yardley S, Teunissen PW, Dornan T (2012). Experiential learning: AMEE guide no. 63. Med Teach.

[44] de Groot E, Endedijk M, Jaarsma D, van Beukelen P, Simons RJ (2013). Development of critically reflective dialogues in communities of health professionals. Adv Health Sci Educ Theory Pract.

[45] de Groot E, Endedijk MD, Jaarsma ADC, Simons PR-J, van Beukelen P (2014). Critically reflective dialogues in learning communities of professionals. Stud Contin Educ.

[46] Snow DA, Benford RD (1988). Ideology, frame resonance, and participation mobilization. Int Soc Mov Res.

